# Analysis of the effect of changes in serum osteopontin levels on patients with acute cerebral infarction

**DOI:** 10.12669/pjms.40.4.7045

**Published:** 2024

**Authors:** Ying Zhang, Jia-rong Wang, Er-na Zhang, Zhi-jiang Zhao

**Affiliations:** 1 Ying zhang, Department of Neurology, Baoding No.1 Hospital, Baoding, 071000, Hebei, P.R. China; 2 Jia-rong Wang, Department of Oncology, Baoding Children’s Hospital, Baoding, Hebei, 071051, P.R. China; 3 Er-na Zhang, Department of Neurology, Baoding No.1 Hospital, Baoding, 071000, Hebei, P.R. China; 4 Zhi-jiang Zhao, Department of Neurology, Baoding No.1 Hospital, Baoding, 071000, Hebei, P.R. China

**Keywords:** Acute Cerebral Infarction, Osteopontin, Neurological Function, Prognosis

## Abstract

**Objective:**

To investigate the correlation of serum osteopontin levels with disease severity and prognosis in patients with acute cerebral infarction.

**Methods:**

This retrospective analysis included forty patients with acute cerebral infarction (ACI) admitted to the Department of Neurology of Baoding Children’s Hospital from May, 2019 to May, 2022 within 24 hours of onset were selected as the observation group, while 40 healthy subjects in our hospital during the same period were selected as the control group. The correlation between serum Osteopontin (OPN) levels and risk factors on one day, seven days and 14 days was analyzed. Patients in the observation group were subdivided into the good prognosis group and the poor prognosis group according to mRS score, and the serum OPN levels of the two groups were compared. The correlation between serum OPN and disease severity and prognosis of patients with ACI was analyzed.

**Results:**

The serum OPN levels in the observation group were significantly higher than those in control group (P< 0.05), and its level was positively correlated with NIHSS score and infarct size. The proportion of patients with hyperlipidemia, smoking, drinking, hypertension and OPN level on seven day in the poor prognosis group were higher than those in the good prognosis group (P<0.05). The OPN level > 8.720 ng/ml on seven days was an independent risk factor for poor prognosis of cerebral infarction.

**Conclusion:**

OPN is involved in the entire pathophysiological process of ACI, and its level can predict the severity of the disease in patients with ACI, and can be used as an important indicator for evaluating their clinical prognosis.

## INTRODUCTION

Currently, among the causes of death due to disease worldwide, stroke is the second leading cause of death after malignant tumors.^1^ About 67% of stroke survivors have varying degrees of disability.^2^ Up to now, there are about seven million stroke patients in China, of which about 65% are ischemic strokes. With the acceleration of the aging society in China, an upward trend is witnessed in the incidence of acute cerebral infarction (ACI). Despite the significant improvement in the diagnosis and treatment of ACI, the disability rate and mortality rate of ACI are still hovering at a high level. To this end, an assessment of the prognostic risk of ACI should be carried out as early as possible and corresponding interventions should be adopted clinically, in order to have a significant impact on the prognosis of patients with ACI.

Osteopontin (OPN), a secreted extracellular matrix protein, acts as a potent soft tissue mineral inhibitor resulting in of inhibiting soft tissue mineralization, delaying and preventing ectopic calcification of the vasculature.^3^ Existing studies have shown that OPN can protect nerves after brain injury.^4^ OPN is also a cytokine involved in the inflammatory response, which intensifies the inflammatory response.^5^ However, few studies have been conducted on the role of OPN in stroke in humans. In this study, the correlation between the changes in OPN level and the prognosis of patients with ACI was explored, providing a reference for clinical practice.

## METHODS

By retrospective analysis, forty patients with acute cerebral infarction (ACI) admitted to the Department of Neurology of Baoding Children’s Hospital from May 2019 to May 2022 within 24 hours of onset were selected as the observation group. In addition, fourty healthy subjects without a history of stroke and myocardial infarction or angina pectoris in our hospital during the same period were selected as the control group.

### Ethical Approval

This study has been approved by the medical ethics committee of Ethical Approval: Baoding No.1 Hospital (No.:2023020703; date: February 28, 2023), and written informed consent was obtained from all participants.

### Inclusion criteria:

- Patients who met the diagnostic criteria of Chinese Guidelines for the Diagnosis and Treatment of Acute Ischemic Stroke(2014 edition).
- Patients confirmed by craniocerebral CT or MRI examination after admission.
- Patients who were hospitalized within 24 hours after onset and did not choose thrombolytic therapy within 6 hours after onset.
- Patients ≥18 years of age.

### Exclusion criteria:

- Patients with a history of stroke; Patients with hemorrhagic stroke.
- Patients with coronary heart disease, heart failure, chronic inflammation, intracranial infection/brain tumor and malignant tumor.
- Patients with liver, kidney and other important organ dysfunction.
- Patients with severe abnormal coagulation function.

The detailed clinical data were collected and related examinations, including ECG, lung CT, blood biochemistry, carotid ultrasound, and head MRI, were performed. Infarct size was calculated by the Pullicino formula [infarct size (CM3) = length × width × height × number of positive layers scanned from MRI/2] according to DWI radiography in MRI.^6^

The degree of neurological deficit was assessed according to the National Institutes of Health Neurological Deficit Score (NIHSS).^7^,^8^ The higher the score, the higher the degree of neurological impairment. The clinical prognosis was assessed 90 day after onset by the modified Rankin Scale (mRS) score. A score of 0-2 points indicates a good prognosis, and 3-6 points indicates a poor prognosis. Enzyme linked immunosorbent assay (elisa) was used to detect serum OPN. All patients were followed up for 6 months and were assessed for recurrence.

### Statistical analysis

All data in this study were analyzed by SPSS 19.0 software. Continuous variables were represented by *χ̅*;±*S* Categorical variables were represented by composition ratio (%). The *c*^2^ test was used to assess inter-group differences in categorical variables, and the independent sample t test was used for inter-group comparisons of continuous variables. Pearson correlation analysis and Logistic regression analysis was used in this study. P<0.05 indicates a statistically significant difference.

## RESULTS

No statistically significant difference was observed between the two groups in general conditions (P>0.05). The number of patients with hypertension, blood pressure, fasting blood glucose, total cholesterol and C-reactive protein in the observation group were significantly higher than those in the control group (P<0.05, Table-I). The serum OPN levels in the observation group were significantly higher than those in the control group on one day and seven days (P<0.05, Table-II).

**Table-I:** Comparison of general conditions between the two groups.

Item	Observation group (n=40)	Control group (n=40)	F/t value	P value
Age (years old)	67.18 ± 6.79	66.55 ± 6.02	0.436	0.664
Gender, male (%)	25 (62.50)	23 (57.50)	0.208	0.648
Diabetes mellitus, number of cases (%)	9 (22.50)	8 (20.00)	0.075	0.785
Hyperlipidemia, number of cases (%)	14 (35.00)	11 (27.50)	0.524	0.469
Smoking, number of cases (%)	19 (47.50)	16 (40.00)	0.457	0.499
Alcohol consumption, number of cases (%)	12 (30.00)	14 (35.00)	0.228	0.633
Hypertension, number of cases (%)	24 (60.00)	15 (37.50)	4.053	0.044
Systolic blood pressure (mmHg)	144.73 ± 4.52	126.90 ± 2.73	21.368	0.000
Diastolic blood pressure (mmHg)	86.38 ± 4.05	80.28 ± 3.67	7.064	0.000
Triglyceride (mmol/L)	1.41 ± 0.63	1.40 ± 0.69	0.076	0.940
Fasting blood glucose (mmol/L)	7.47 ± 1.70	6.46 ± 1.83	2.564	0.012
Total cholesterol (mmol/L)	4.68 ± 0.29	4.35 ± 0.56	3.234	0.002
C-reactive protein (mg/L)	6.80 ± 0.63	2.64 ± 0.50	32.591	0.000

**Table-II:** Comparison of serum OPN levels between the two groups.

Group	OPN (ng/ml)		

	1d	7d	14d
Observation group (n=40)	7.75 ± 0.63	8.83 ± 0.66	5.15 ± 0.55
Control group (n=40)	5.09 ± 0.50	4.94 ± 0.42	4.97 ± 0.34
t value	20.839	31.196	1.685
P value	0.000	0.000	0.096

Pearson correlation analysis showed that the serum OPN levels in the cerebral infarction group on one day and seven days were positively correlated with the NIHSS score and infarct size of the patients, Table-III. According to the mRS results, there were 27 cases (67.50%) in the good prognosis group and 13 cases (32.50%) in the poor prognosis group. The proportion of patients with hyperlipidemia, smoking, drinking and hypertension and the OPN level on seven days in the poor prognosis group were higher than those in the good prognosis group (P<0.05, Table-IV).

**Table-III:** Correlation of OPN level with NIHSS score and infarct size in cerebral infarction group.

OPN level	NIHSS score		Infarct size	

	r	P	r	P
1d	0.873	0.000	0.397	0.011
7d	0.406	0.009	0.314	0.048
14d	-0.139	0.392	-0.003	0.987

**Table-IV:** Comparison of clinical data of patients with cerebral infarction with different prognosis.

Item	Good prognosis with mRS score (n=27)	Poor prognosis with mRS score (n=13)	F/t value	P value
Age (years old)	67.52 ± 6.04	66.46 ± 8.37	0.456	0.651
Gender, male (%)	16 (59.26)	9 (69.23)	0.372	0.542
Diabetes mellitus, number of cases (%)	4 (14.81)	5 (38.46)	2.814	0.093
Hyperlipidemia, number of cases (%)	6 (22.22)	8 (61.54)	5.962	0.015
Smoking, number of cases (%)	16 (59.26)	3 (23.08)	4.607	0.032
Alcohol consumption, number of cases (%)	11 (40.74)	1 (7.69)	4.564	0.033
Hypertension, number of cases (%)	13 (48.15)	11 (84.62)	4.862	0.027
OPN level on 1d	7.63 ± 0.54	8.00 ± 0.75	1.804	0.079
OPN level on 7d	8.70 ± 0.60	9.57 ± 1.09	3.276	0.002
OPN level on 14d	5.04 ± 0.39	5.37 ± 0.77	1.803	0.079

**Fig.1:**
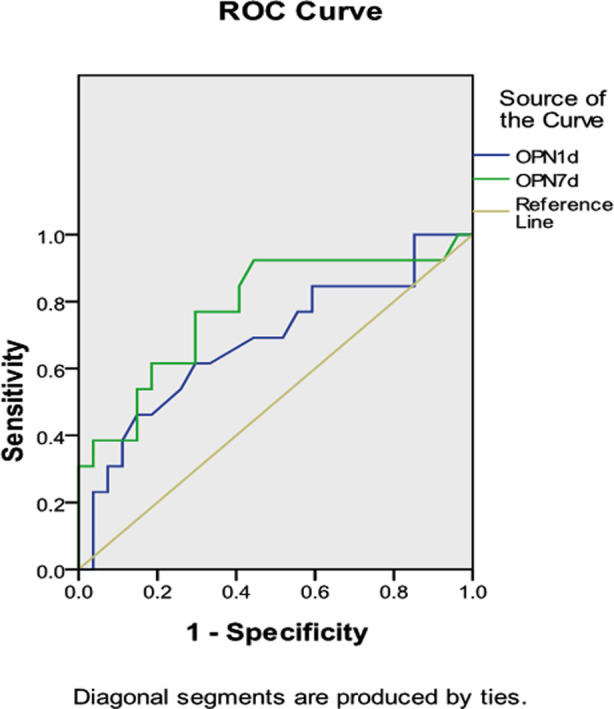
ROC curve analysis

ROC curve showed that the cut-off point was 7.585 ng/ml at the OPN level on day one and 8.720 ng/ml at the OPN level on seven days, as shown in Fig.1. Logistic multiple step-wise regression analysis showed that hyperlipidemia, hypertension and OPN levels on seven days were independently associated with poor clinical outcome and could be used as independent risk factors for the prognosis of cerebral infarction. Table-V.

**Table-V:** Logistic regression analysis of mRS score outcomes in patients with cerebral infarction.

Variable	B	S.E.	wald	Exp(B)	95%C.I.		P

					Lower limit	Upper limit	
Hyperlipidemia	2.403	1.171	4.208	11.051	1.113	109.723	0.040
Hypertension	2.998	1.267	5.602	20.051	1.674	240.128	0.018
OPN level on 7d	3.691	1.437	6.600	40.092	2.399	669.941	0.010

## DISCUSSION

In this study, the relationship between OPN level at the acute stage of ACI and different neurological impairment and prognosis was analyzed. The results showed that OPN level was positively correlated with NIHSS score and infarct size, suggesting that OPN level at the acute stage of ACI could reflect neurological impairment and prognosis, and may be used for neurological impairment and prognosis assessment.

Osteopontin(OPN) is a multifunctional cellular matrix protein widely distributed in the human body, which also exists in the extracellular matrix of nerve tissue, acting as an important repair agent for neural tissue damage.^9^,^10^ According to studies, OPN can regulate the migration of neuroblasts in the subventricular region after brain ischemia.^11^ OPN has also been found to be an enhancer of atherosclerosis, and its level is significantly associated with adverse cardiac events and an increased risk of coronary atherosclerosis.^12^ It was considered by Kurata et al. that OPN could be used as a biomarker for thrombosis in patients with acute cerebral ischemia.^13^ Similar to most clinical studies, our study showed that the dynamic changes in OPN level in patients with ACI generally reached a peak about 5-7 days after onset, which was statistically significant compared with the control group. After 14 day, the OPN level in patients with cerebral infarction basically returned to normal. It shows that OPN is involved in the pathogenesis, nerve injury and deterioration of acute cerebral infarction, and has a certain impact on the degree of neurological impairment and prognosis of patients with acute cerebral infarction.

Osteopontin (OPN) can promote the accumulation of mononuclear macrophages in the inflammatory area, and at the same time regulate the production of various cytokines, thereby participating in the inflammatory response of the body’s blood vessels.^14^ It was found in a study^15^ that in vascular diseases, if OPN is sharply increased, it can reduce vascular calcification and promote the formation of ischemic local neovascularization and a series of protective effects. Studies have shown^16^ that interleukin-L α and platelet-derived growth factors can induce THE expression of OPN, leading to the progression of atherosclerosis. This study also suggested that OPN is an enhancer of atherosclerosis, and that OPN level is associated with an increased risk of adverse events in cardiovascular and cerebrovascular acute infarction. It has also been demonstrated in other studies^17^ that OPN is an independent risk predictor of cardiovascular events in patients with atherosclerotic heart disease treated with percutaneous coronary intervention.

Logistic multiple regression analysis showed that hyperlipidemia, alcohol consumption, hypertension and OPN level on sevenrth day had predictive effects on the poor prognosis of patients with acute cerebral infarction. Our study suggests that OPN promotes atherosclerotic plaque formation through the following three possible mechanisms^18^-^20^:

- OPN down-regulates the calcification degree of atherosclerotic plaques, leading to plaque instability.
- OPN induces the release of protease substances from atherosclerotic plaque, and promotes the formation of neovascularization in plaque, leading to plaque bleeding and fibrous cap rupture.
- Most of the history of high blood pressure patients suffer from alcohol, hyperlipidemia. These three factors complement and promote each other, leading to atherosclerosis and finally acute cerebral infarction.

### Limitations

All patients were recruited from the investigator’s hospital with a small sample size, and there may be selection bias. Moreover, the OPN level of the subjects was only monitored three times in this study, and no monitoring was done for OPN levels during post-discharge follow-up. Therefore, further research is needed to determine whether the level of OPN is positively correlated with size and scales of cerebral infarction.

## CONCLUSION

In conclusion, we confirmed in this study that the level of OPN is positively correlated with the size and severity of acute cerebral infarction, and has an obvious prognostic effect on acute cerebral infarction.

### Authors’ Contributions:

**YZ and JW** carried out the studies, participated in collecting data, drafted the manuscript, are responsible and accountable for the accuracy and integrity of the work.

**EZ** performed the statistical analysis and participated in its design.

**ZZ** participated in acquisition, analysis, or interpretation of data and draft the manuscript.

All authors read and approved the final manuscript.
